# Modulation of Recombinant Antigenic Constructs Containing Multi-Epitopes towards Effective Reduction of Atherosclerotic Lesion in *B6;129S-Ldlr^tm1Her^Apob^tm2Sgy^/J* Mice

**DOI:** 10.1371/journal.pone.0123393

**Published:** 2015-04-01

**Authors:** Min Xia, Daxin Chen, Valeria Endresz, Ildiko Lantos, Andrea Szabo, Vijay Kakkar, Xinjie Lu

**Affiliations:** 1 The Mary and Garry Weston Molecular Immunology Laboratory, Thrombosis Research Institute, London, United Kingdom; 2 MRC Centre for Transplantation, King's College London, London, United Kingdom; 3 Department of Medical Microbiology and Immunobiology, University of Szeged, Szeged, Hungary; 4 Institute of Surgical Research, University of Szeged, Szeged, Hungary; 5 Thrombosis Research Institute, Bangalore, India; Max-Delbrück Center for Molecular Medicine (MDC), GERMANY

## Abstract

Atherosclerosis is increasingly recognized as a complex chronic inflammatory disease. Many more studies have extended vaccination against atherosclerosis by using epitopes from self-antigens or beyond and demonstrated that vaccination with antigens or derivatives could reduce the extent of the lesions in atherosclerosis-prone mice. Our previous study has demonstrated that construct AHHC [ApoB100^688-707^ + hHSP60^303-312^ + hHSP60^153-163^ + Cpn derived peptide (C)] significantly reduced atherosclerotic lesion. The aim of this study was to investigate whether AHHC can be modulated towards increased lesion reduction in mice by creating two other derivatives with a sequential epitope-substitution named RHHC in which A was replaced by an “R” (C5aR^1-31^) and RPHC with a further “H” (hHSP60^303-312^) conversion into “P” (protease-activated receptor-1^42-55^) in mice. Antigenic epitopes were incorporated into a dendroaspin scaffold. Immunization of B6;129S-Ldlr^tm1Her^Apob^tm2Sgy^/J mice with three constructs elicited production of high levels of antibodies against each epitope (apart from hHSP60^153-163^ and P which induced a low antibody response). Histological analyses demonstrated that the mice immunized with either RPHC or RHHC showed significant reductions in the size of atherosclerostic lesions compared to those with AHHC (69.5±1.1% versus 55.7±3.4%, *P*<0.01 or 65.6±1.3% versus 55.7±3.4%, *P*<0.01). Reduction of plaque size in the aortic sinus and descending aorta correlated with alterations in cellular immune responses when compared with controls. We conclude that a recombinant construct RPHC may provide new antigenic and structural features which are favorable for significant reduction in atherosclerotic lesion formation. This approach offers a novel strategy for developing anti-atherosclerotic agents.

## Introduction

Atherosclerosis is increasingly recognized as a complex chronic inflammatory disease of the arterial walls [1–3] as evidenced by the presence of inflammatory cells, activated immune cells and cytokines in lesions, all of which indicate involvement of the immune system [4,5]. Dendritic cells (DCs) are likely to play a crucial role in directing innate and adaptive immunity. A major component of the innate response involves the entry of monocytes into nascent lesions, followed by differentiation of monocytes into macrophages and CD11c^+^ cells with DC-like properties [6]. Atherosclerotic plaques are known to contain macrophage-derived foam cells in which macrophages interact with T cells to produce a wide array of cytokines that can exert both

pro- and anti-inflammatory effects [7,8]. Although the molecular mechanism responsible for the development of atherosclerosis is not completely understood, it is clear that the immune system plays a key role in the development of the atherosclerotic plaque and in its complications. Consequently, several antigenic stimuli that are associated with the pathogenesis of atherosclerosis based on modified self-molecules or peptides derived from these molecules such as oxidized low-density lipoproteins (oxLDLs) [9,10], β2-glycoprotein I (β2GP1), phosphatidylcholine (PC) [11,12], heat shock proteins (HSPs), [13] have been reported. Many more studies related to vaccination against atherosclerosis apart from using epitopes from these self-antigens have also demonstrated high efficacy against atherosclerotic lesion formation for other antigens [14,15]. However, one of the difficulties in developing effective vaccination strategies against atherosclerosis is the selection of a specific antigen. In the discovery and development of potential antigens, we have previously demonstrated that immunization of *B6;129S-Ldlr* ^*tm1Her*^ *Apob* ^*tm2Sgy*^ */J* mice with the recombinant construct AHHC containing epitopes derived from apolipoprotein B (ApoB), heat shock protein (HSP) 60 and proteins of *Chlamydia pneumoniae* (Cpn), significantly reduced atherosclerotic lesion formation in the mice fed with high-fat diet (HFD) [16]. In addition, we have also demonstrated that immunization with peptides derived from the N-terminal of complement component 5a receptor (C5aR) significantly reduced early atherosclerotic lesion development in *B6;129S-Ldlr* ^*tm1Her*^ *Apob* ^*tm2Sgy*^ */J* mice [17]. Furthermore, in preclinical studies, protease-activated receptor (PAR)-1 inhibition showed a strong antithrombotic effect, leading to a significant decrease in platelet aggregation, whereas primary haemostatic function was preserved [18] and our recent study in *B6;129S-Ldlr* ^*tm1Her*^ *Apob* ^*tm2Sgy*^ */J* mouse model showed that PAR-1 peptide significantly attenuated atherosclerotic lesion formation[19]. Based on the effects of the peptides derived from C5aR and PAR-1 on reducing the atherosclerotic lesion, we hypothesized that the effect of a multi-epitopic construct on reducing atherosclerotic lesion may be modulated towards favorable plaque phenotype and increased lesion reduction with inclusion of C5aR and PAR-1 in vaccination. In the present study we investigated the effect of C5aR and PAR-1 including constructs through a sequential substitution: AHHC→RHHC (R denotes an epitope derived from C5aR) in RHHC→RPHC (P denotes an epitope derived from PAR-1) on reducing atherosclerotic lesion.

## Methods

### 1. Expression and purification of recombinant glutathione S-transferase constructs

Glutathione S-transferase-dendroaspin (GST-Den here referred to as a control, S1A Fig) and GST-recombinant construct AHHC [ApoB100 peptide, amino acids (aa) 688–707 (numbered including signal peptide), human heat shock protein (hHSP) 60 peptide, aa 303–312 and aa153-163, respectively; and *Chlamydia pneumoniae* (Cpn) derived epitope (“C”, denotes a combination of aa 66–73 from major out membrane protein (MOMP) and aa 283–291 from outer membrane 5 of Cpn)] was described previously and construct RHHC (“R” denotes a C5aR peptide, aa 1–31) and RPHC (“P” denotes an protease activated receptor-1 (PAR-1) peptide, aa 42–55) were generated. Schematic presentation of these constructs using dendroaspin as a scaffold is shown in S1B Fig These recombinant molecules were expressed in *Escherichia coli* (BL-21 strain), purified by affinity and ion exchange chromatography and analyzed by sodium dodecyl sulfate polyacrylamide gel electrophoresis, all these procedures were similar to those described for AHHC previously [16].

### 2. Animal experiments

The experiments were approved by the Animal Welfare Committee of the University of Szeged and conform to the Directive 2010/63/EU of the European Parliament.

Low-density lipoprotein receptor–deficient apolipoprotein B-100-only mice were used in our study, each group of mice consisting of 5-6-week-old males. We used dendroaspin as a control antigen, since dendroaspin was used as a scaffold and our previous data showed no effect of dendroaspin on lesion reduction when used for subcutaneous immunization in mice [16].

The immunizing antigens used were constructs AHHC, RHHC and RPHC, respectively. The repetitive immunization multiple sites strategy (RIMMS) was adopted [1] and mice were sacrificed at the end of week 12 (a high-fat diet was started at the end of week 2 and continued for 10 weeks). The control groups followed the diet program after immunization with GST-tagged Den and Alum (adjuvant).

### 3. Tissue preparation and antibody response measurements

Twelve weeks after the first immunization, aorta tissues were harvested and mounted in optimal cutting temperature compound (OCT) and in paraffin, for immunohistochemical (IHC) analyses and lesion measurement, respectively. Atherosclerotic lesions in aortic roots were examined by an Olympus UULH Optical microscope (Olympus Optical Co. Ltd, Tokyo, Japan) and analyzed with Image-Pro Plus software, version 7.0 (Media Cybernetics, Inc., Bethesda, MD, USA). Longitudinally opened descending aortas were evaluated for the extent of atherosclerosis after Oil Red O (ORO) staining. The peptide-specific antibody levels in the plasma samples were measured by ELISA following the manufacturer’s instructions. One third of spleens were embedded in OCT and the remaining part was homogenized by pressing through 70 μm cell strainer and frozen for further analysis.

### 4. IHC and morphometric analyses, quantitative measurements of atherosclerosis, and IHC analysis of forkhead box protein 3 (Foxp3) expression in CD4^+^ splenocytes

OCT-embedded samples were used for detection of CD68, CD11c, Interleukin (IL)-10 and tumor necrosis factor (TNF)-α, Foxp3, vascular cell adhesion molecule (VCAM)1, alpha smooth muscle cell (alpha-SMC), matrix metalloproteinase 9 (MMP9) by IHC analyses. Sections of paraffin-embedded tissues were stained with hematoxylin and eosin (HE) and elastin/van Gieson (Sigma) for histological examination by the Olympus U-ULH Optical microscope.

### 5. Flow cytometric analysis of Foxp3, IL-2, IL-4 and IL-17A expression in CD4^+^ T-cells in splenocytes and differentiation of PBMC into macrophage (CD206)

Spleen cells were processed for staining (30 min at 4°C) using allophycocyanin-anti-mouse Foxp3, IL-2, IL4 and IL-17 antibodies (BioLegend, Cambridge, UK). For cell differentiation assay, mouse (C57BL/6) PBMCs were stimulated with different antigens or pre- incubated with antiserum of antigen (in order to see any antagonism of antiserum). Antigen-induced differentiation of monocytes into macrophages was measured by flow cytometry which was compared to cell populations from non-induced cells or control antigen (GST-Den)-induced cells.

### 6. Measurement of cytokines

IL-10 and TNF-α levels in the lesions were quantified by IHC analyses (rat anti-mouse TNF-α and IL-10 purchased from BioLegend, CA, USA). Plasma levels of the cytokines, IL-10, transforming growth factor beta (TGF-β), TNF-α and interferon gamma (IFN-γ) were measured by ELISA following the manufacturer’s instructions (R&D systems, Abingdon, UK). Levels of concanavalin A (ConA)-induced IL-10, TGF-β, TNF-α, and IFN-γ in splenocyte cultures were also measured.

### 7. Antigen-specific regulatory T cell function assays

To assess antigen-specific regulatory T cell function, CD4^+^CD25^+^ Treg cells were isolated by using regulatory T cell isolation kit of Miltenyi Biotec (Bergisch Gladbach, Germany) from spleen CD4^+^ T cells of *B6;129S-Ldlr* ^*tm1Her*^ *Apob* ^*tm2Sgy*^ */J* mice immunized subcutaneously with constructs, respectively. CD4^+^CD25^−^ T effector cells were isolated from spleen CD4^+^ T cells (unbound to beads binding CD4^+^CD25^+^ cells, 99.5% of CD4^+^ cells) of same construct-immunized mice respectively. CD4^+^CD25^−^ cells (2×10^5^) were co-cultured with CD4^+^CD25^+^ cells (2×10^5^), and stimulated with 1 μM related construct or with GST-Den control. After 2 days of culture, the proliferation of T effector cells was measured for the shift in fluorescence intensity of a population of cells by flow cytometry expressed as mean fluorescence intensity (MFI).

### 8. Statistical analyses

Data are reported as mean±standard error of the mean (±SEM), unless otherwise indicated. Figures were plotted using graph-pad Prism 5.01 and Sigma plot 9.0. For atherosclerotic lesion size, data were compared and intergroup differences were analyzed using one-way ANOVA for multiple comparisons and post hoc Bonferroni test. Other data were analyzed using Student’s t-test (2-tailed analyses). Non-parametric distributions were analyzed using the Mann-Whitney U test for pair wise comparisons and the Kruskal-Wallis test for multiple comparisons. Differences between groups were considered significant at *P* values below 0.05. An extended description is available as S1 Text, S1 Table, S1 Fig, S2 Fig, S3 Fig, S4 Fig, and S5 Fig.

## Results

### 1. Peptide-specific immunoglobulin G in the sera of immunized mice

**Fig 1:**
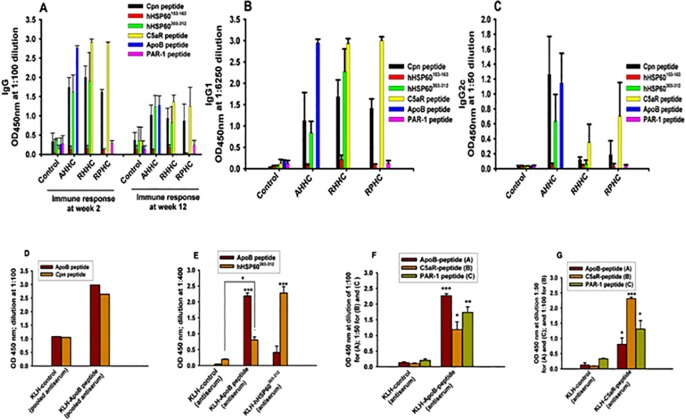
Levels of constructed protein-induced IgG, IgG1, and IgG2c antibodies in the sera of *B6;129S-Ldlr* ^*tm1Her*^ *Apob* ^*tm2Sgy*^ */J* mice at 2 weeks and 12 weeks respectively, after the first immunization and in controls (GST-Den-immunized mice). The mean optical densities (ODs) and SEM obtained from plasma samples of constructs AHHC, RHHC, and RPHC-immunized mice on Cpn peptide (C)-, hHSP60^153-163^ (H)-, hHSP60^303-312^ (H)-, C5aR-peptide (R)-, ApoB peptide (A)-, PAR-1(P)-coated ELISA plates are shown. (A) IgG at the dilution ratio: 1:100. (B) IgG1 at the dilution ratio: 1:6250. (C) IgG2c at the dilution ratio: 1:50. (D) Cross reaction between human ApoB peptide-induced antiserum and Cpn peptide. (E) Cross reaction between human ApoB peptide- and hHSP60^303-312^ peptide-induced antiserum and their antigens. (F) Cross reaction between ApoB peptide-induced antiserum and antigens of either PAR-1 peptide or C5aR peptide. (G) Cross reaction between C5aR peptide-induced antiserum and antigens of either ApoB peptide or PAR-1 peptide. Antiserum used for cross-reaction was taken at week 8.

Antibody levels were measured by ELISA test in the sera of mice immunized with either dendroaspin (GST-tagged) or constructs within dendroaspin scaffold (GST-tagged) at weeks 2 and week 12 respectively after first immunization. In AHHC-immunized mice, ApoB peptide-, hHSP60^303-312^- and Cpn-peptide-specific antibodies were observed when these peptides were used as ELISA antigens (Fig 1A). Similarly C5aR peptide-specific antibodies were detected in addition to observed hHSP60^303-312^- and Cpn peptide- specific antibodies in mice immunized with RHHC (Fig 1A). High antibody levels against Cpn peptide and C5aR peptide whereas low antibody level against PAR-1 peptide was detected in mice immunized with RPHC (Fig 1A). Low antibody level against hHSP60^153-163^ was observed in mice immunized with any construct (Fig 1A). Overall observed optical density (OD) values at 100 dilutions were reduced at week 12 compared to those at week 2.

Interestingly, a peptide-induced specific immunoglobulin (Ig)G1 response was observed in serum of peptide-immunized mice against all peptide antigen epitopes at high dilutions except for those against hHSP60^153-163^ and PAR-1 peptides when compared with GST-Den control (Fig 1B). In addition, a similar pattern for IgG2c response was also detected but with low serum dilutions when compared with that in control (Fig 1C). However, the levels of IgG2c detected were much lower than those of IgG1 based on the optical densities measured in different dilutions of samples (1:50 versus 1:6250). Furthermore, using antiserum at week 8, certain levels of cross-reactions were observed between ApoB peptide-induced antiserum and Cpn peptide (Fig 1D), between ApoB peptide- and hHSP60^303-312^ peptide- induced antiserum and their antigens (Fig 1E), between ApoB peptide-induced antiserum and antigens of either PAR-1 peptide or C5aR peptide (Fig 1F) and between C5aR peptide-induced antiserum and antigens of either ApoB peptide or PAR-1 peptide (Fig 1G). Apart from cross-reaction between Cpn peptide and ApoB peptide antiserum when pooled sera were tested and thus no SD values were calculated (Fig 1D), the other cross-reactions showed significant difference compared to the controls (Fig 1E–1G; *P*<0.05~<0.001).

### 2. Reduction of atherosclerotic lesion size in the aortic sinus

**Fig 2:**
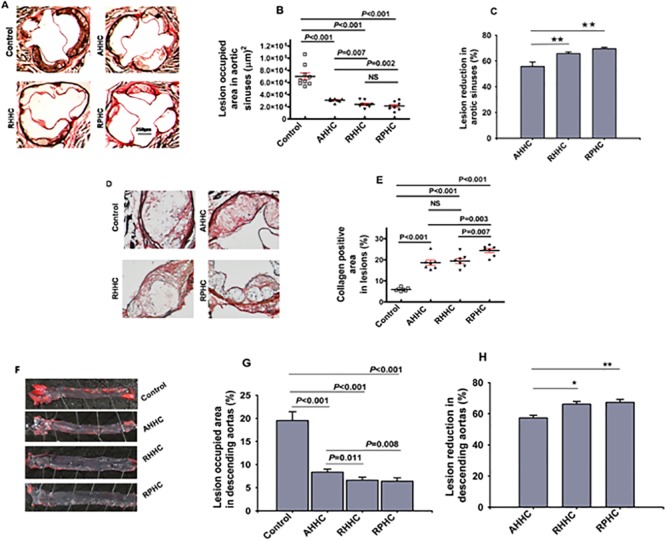
Detection and quantitation of the lesion areas in the aorta of *B6;129S-Ldlr* ^*tm1Her*^ *Apob* ^*tm2Sgy*^ */J* mice fed on a high-fat diet after immunization with constructs versus controls (GST-Den). (A) Photomicrograph of lesions observed in atherosclerotic aortas as analyzed with elastin/van Gieson staining. (B) Scatter plot showing mean of lesion area in the aortic sinus of mice immunized with constructs compared with those in controls (GST-Den). (N = 6–9 mice). Error bars = SEM. (C) Percentage of reduction in lesion size in the aortic sinus (the reduction of control [GST-Den] group was set at zero). (D) Representative photomicrographs and quantitative analysis of collagen (Sirius Red coloration under polarized light) in atherosclerotic aortas in individual mice. (E) Quantification of collagen content at lesion area in the aorta of *B6;129S-Ldlr* ^*tm1Her*^ *Apob* ^*tm2Sgy*^ */J* mice (N = 7 mice). NS: not significant. (F) Representative Oil Red O-stained *en face* descending aorta from mice. (G) Percentage of lesion-occupied area versus total area of descending aortas in individual mice (N = 6 mice) of the different experimental groups. The mean lesion size and the difference in lesion size between the experimental groups are shown. (H) Percentage of reduction in lesion size in descending aortas (the reduction of control [GST-Den] group was set at zero).

Following immunization with AHHC, RHHC, RPHC, and after a 10-week high-fat diet, the aortic sinuses of the mice were evaluated for the extent of atherosclerosis. The calculated plaque sizes from the immunized animals were compared with those of the controls. Representative photomicrograph of sections with lesions from experimental groups are shown in Fig 2A. The plaque areas are shown in Fig 2B. Lesion size was smaller in mice immunized with all three constructs showing 31,071 ± 998.7 μm^2^, 24,123 ± 1967μm^2^ and 21,386 ± 2482 μm^2^ (*P*<0.001) compared with controls (70,200 ± 5718 μm^2^). The smaller lesion area was observed in mice immunized with either RHHC or RPHC compared with mice immunized with AHHC (*P* = 0.007–0.002) (Fig 2B). The percentage of reduction in lesion size is shown in Fig 2C, showing 55.7 ± 3.4%, 65.6 ± 1.3% and 69.5 ± 1.1% when assuming reduction in lesion of control animals as zero percent. The control mice that were immunized with GST-Den showed similar lesion formation in either GST-tag-or Alum (adjuvant)-immunized controls (S2A and S2B Fig). Therefore, we took GST-Den as a control throughout the experiment.

In addition, we examined the impact of treatment with these recombinant constructs on the collagen content in these lesions. The reduction of atherosclerosis in mice treated with these constructs was associated with an increase in collagen content: approximately 3-fold for either AHHC-immunized mice or RHHC-immunized mice versus control mice (18.6 ± 1.2% or 19.4 ± 0.9% versus control 5.9 ± 0.3%; *P<0*.*001*), respectively (Fig 2D and 2E). Mice immunized with in RPHC showed a significant collagen increase (24.4 ± 0.9%) compared with mice immunized with either AHHC or RHHC (*P =* 0.003, and *P =* 0.007, respectively).

Longitudinally opened descending aortas were stained en face with oil red O (ORO) and positively stained plaques areas were measured. Representative en face stained descending aortas from experimental groups are shown in Fig 2F. Lesion size was significantly smaller in mice immunized with any construct showing 8.4 ± 0.3%, 6.6 ± 0.3% and 6.4 ± 0.4% for AHHC, RHHC, and RPHC, respectively compared with controls (19.5 ± 1.7%, *P*<0.001) (Fig 2G). Both RHHC- and RPHC-immunized mice showed significantly less lesion than AHHC-immunized mice (*P* = 0.011–0.008). The reduction of lesions expressed as a percentage is shown in Fig 2H with 57.3 ± 1.7%, 66.1 ± 1.6% and 67.3 ± 1.9% for AHHC, RHHC, RPHC, respectively. The smallest lesion area was observed in mice immunized with RPHC.

### 3. Amount of inflammatory cells and CD4^+^ T cells expressing Foxp3 in local (lesions of aortas) and remote organs: splenocytes and lymphocytes

**Fig 3:**
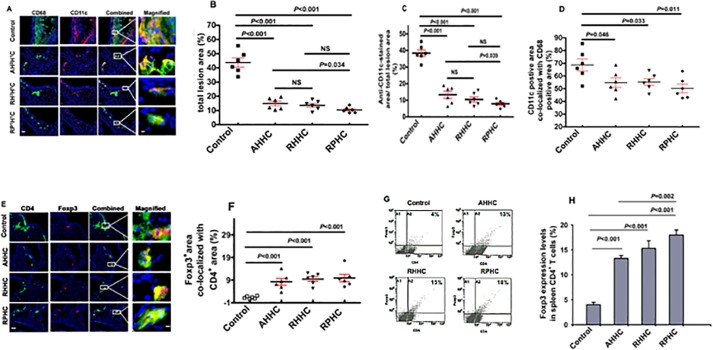
Assessment of inflammation-associated cells in the lesions of *B6;129S-Ldlr* ^*tm1Her*^ *Apob* ^*tm2Sgy*^ */J* mice mice fed a high-fat diet after immunization with constructs AHHC, RHHC, and RPHC. (A) Photomicrographs showing IHC staining of CD68 (green) and CD11c (red) markers (scale bar: 100 μm and 12.5μm for magnified ones). (B) Scatter plot showing anti-CD68-stained area in lesion versus total lesion area; Data are given as the mean of 6 mice. (C) Scatter plot showing anti-CD11c-stained area in lesion versus total lesion area; Data are given as the mean of 6 mice. (D) Co-localization of CD68^+^ and CD11c^+^ areas (derived from Fig 3B and 3C). (E) Photomicrographs showing IHC staining of CD4^+^ T-cells (green) and Foxp3^+^ Treg cells (red) (scale bar: 100 μm and 12.5μm for magnified ones). (F) Scatter plot showing anti-Foxp3-stained area versus anti-CD4^+^ stained area in lesion (N = 6). (G) Representative analysis of Foxp3 expression by CD4^+^ T cells in lymph nodes from construct-immunized mice fed on a high-fat diet as assessed using flow cytometry. (H) Percentage of Foxp3^+^ cells among CD4^+^ spleen cells as analyzed by flow cytometry. Data are expressed as mean of 3 analyses ± SEM. Differences between groups are shown.

The percentage of anti-CD68-stained area in the lesions showed 14.9 ± 1.6%, 13.6 ± 1.3% and 10.3 ± 0.8% in mice immunized with AHHC, RHHC and RPHC, respectively compared to 43.7 ± 3.2% in control mice immunized with GST-Den (*P*<0.001). Lesser anti-CD68-stained area was observed in RPHC-immunized mice compared with that in AHHC-immunized mice (*P* = 0.034) (Fig 3A and 3B). Similarly, measurement of anti-CD11c-stained lesion area showed 13.3 ± 2.1%, 10.5 ± 1.5% and 7.9 ± 0.8% in mice immunized with AHHC, RHHC and RPHC, respectively compared to 38.4 ± 1.9% in control mice (*P*<0.001) (Fig 3A and 3C). Anti-CD11c-stained lesion area in RPHC-immunized mice was significantly smaller when compared with that in AHHC-immunized mice (*P* = 0.039) (Fig 3A and 3C). Double immunostaining for CD68 and CD11c clearly revealed that more than half of macrophages were CD68^+^CD11c^+^, indicating the myeloid origin of this cell type in lesions as CD11c^+^ area co-localized with CD68^+^ area expressed as percentage showing 54.7 ± 3.7%, 55.2 ± 2.6% and 50.3 ± 3.3% for AHHC, RHHC and RPHC, respectively compared to 68.6 ± 4.7% in control mice (Fig 3A–3D; *P* = 0.046–0.011) [20].

The proportion of CD4^+^ cells expressing Foxp3 analyzed by IHC staining of aorta sections was approximately 6- to 8-fold higher (8.2 ± 1.4%, *P*<0.001; 9.4 ± 1.1%, *P*<0.001; 9.9 ± 1.6%, *P*<0.001) in mice immunized with AHHC, RHHC and RPHC, respectively compared to 1.2 ± 0.2% in control mice (*P*<0.001) (Fig 3E and 3F). In addition, expression of Foxp3 in CD4^+^ spleen cells from mice immunized with these three constructs was higher (*P*<0.001) than that in controls, showing 13.3 ± 0.6%, 15.3 ± 1.5%, and 18.0 ± 1.1%, for AHHC, RHHC and RPHC respectively versus 4.0 ± 0.5% in controls (Fig 3G and 3H). With the exception of RPHC-immunized mice, which did not show a significant increase of Foxp3 expression when compared with that in RHHC-immunized mice, however, significantly higher levels of Foxp3 expression were observed in comparison with that in AHHC-immunized mice (*P* = 0.002).

### 4. Expression of anti-inflammatory cytokines and proinflammatory cytokines in lesion sites and levels of cytokines in plasma and in the supernatants of stimulated splenocytes

**Fig 4:**
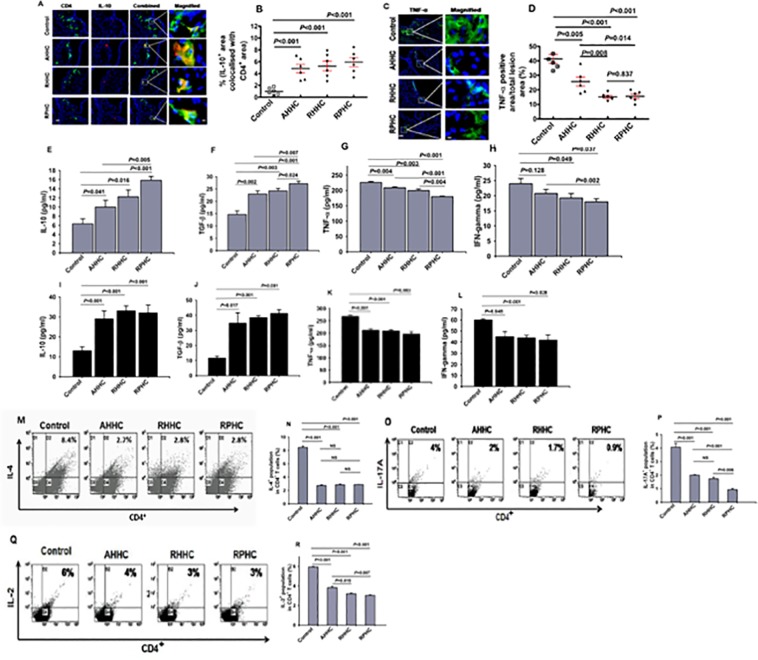
Assessment of IL-10-producing T cells, TNF-α expression in the lesions and cytokine levels in *B6;129S-Ldlr* ^*tm1Her*^ *Apob* ^*tm2Sgy*^ */J* mice fed a high-fat diet after immunization with constructs AHHC, RHHC, and RPHC. (A) Photomicrographs showing dual-IHC staining for IL-10 (red) and CD4 (green) (scale bar: 100 μm and 12.5μm for magnified ones). (B) Scatter plot showing mean of IL-10-positive area co-localized with CD4^+^ area (%) (N = 6). (C) Photomicrographs showing IHC staining for TNF-α (green) of lesions (scale bar: 100 μm and 12.5μm for magnified ones). (D) Scatter plot showing mean of anti- TNF-α-stained area in the lesion versus total lesion area (N = 6). (E-H) Cytokine levels measured in plasma. (I-L) Cytokine levels measured in the supernatant of splenocytes stimulated with ConA. (M) Representative analysis of CD4^+^IL-4^+^ T-cells in splenocytes from construct-immunized mice fed on a high-fat diet as assessed by flow cytometer. (N) Percentages of IL-4^+^ expressing CD4^+^ spleen cells. Data are expressed as mean of 3 analyses ± SEM. Differences between groups are shown. (O) Representative analysis of IL-17A expression by CD4^+^ T-cells in splenocytes from construct-immunized mice fed on a high-fat diet as assessed by flow cytometry. (P) Levels of IL-17A expression in spleen cells. Data are expressed as mean of 3 analyses ± SEM. Differences between groups are shown. (Q) Representative analysis of CD4^+^IL-2^+^ T-cells in splenocytes from construct-immunized mice fed on a high-fat diet as assessed by flow cytometer. (R) Levels of IL-2^+^ expressing CD4^+^ in spleen cells. Data are expressed as mean of 3 analyses ± SEM. Differences between groups are shown.

IL-10 expression in the aortic lesions of mice immunized with AHHC, RHHC and RPHC, detected by IHC analyses is shown in Fig 4A. The proportion of CD4^+^ cells expressing IL-10 in the lesions was approximately 6-fold higher in mice immunized with these constructs, showing 4.8 ± 0.7%, 5.2 ± 0.8% and 5.9 ± 0.7% (*P*<0.001) for AHHC, RHHC and RPHC, respectively when compared with that in the control group (0.9 ± 0.2%) as shown in Fig 4A and 4B. IHC analyses of TNF-α expression showed significantly smaller TNF-α-occupied areas in lesions of mice immunized with constructs compared with controls (25.7 ± 3.1% for AHHC; 15.2 ± 0.9% for RHHC; 15.5 ± 1.5% for RPHC and 41.3 ± 3.1% for controls) (Fig 4C and 4D). These data represent a percent reduction of 37.8%, 63.2%, and 62.5%, respectively, compared with controls (total lesion area defined as 100% and 0% reduction). Additionally, enhanced reduction was produced by either RHHC or RPHC versus AHHC (*P* = 0.008–0.014).

Plasma levels of atheroprotective cytokines IL-10 were significantly increased in mice immunized with these three constructs compared with controls (Fig 4E and 4F). Immunization with RPHC had more effect than with other two constructs on promoting the secretion of IL-10 and TGF-β (*P*<0.05 to <0.001). Plasma levels of the atherogenic cytokine TNF-α were significantly reduced by immunization with these three constructs (Fig 4G). A similar trend was obtained for these constructs in respect of plasma levels of IFN-γ (Fig 4H). Notably, immunization with RPHC had more effect than with AHHC on reducing the secretion of TNF-α and IFN-γ (*P*≤0.002) and more effect than with RHHC on reducing the secretion of TNF-α (*P* = 0.004). Although slightly higher plasma levels of IFN-γ from mice immunized with AHHC was observed compared to that of control mice (24 versus 20.8 pg/ml), this difference did not show statistical significance.

Supernatants of splenocytes from mice immunized with these constructs individually showed significantly increased secretion of IL-10 (Fig 4I) and TGF-β (Fig 4J), stimulated with 10 μg/mL of ConA (*P*<0.05–0.001) when compared to those of controls. In addition, higher levels of either IL-10 or TGF-β were produced by splenocytes of RPHC-immunized mice than those of AHHC-immunized mice (*P*<0.05–0.01). In contrast, TNF-α (Fig 4K) and IFN-γ levels (Fig 4L) were significantly decreased in the supernatants of splenocytes of mice immunized with these constructs compared with the control when stimulated with 10 μg/mL of ConA. Notably, levels of TNF-α and IFN-γ in RPHC-immunized mice were lower than those in AHHC-immunized mice (*P*<0.05–0.01) when stimulated with 10 μg/mL of ConA, respectively. Interestingly, supernatants of splenocytes from mice immunized were also shown to contain increased amount of protective cytokine IL-10 and decreased amount of proinflammatory cytokine IFN-γ when stimulated with either peptides or construct containing peptides but not in the case when stimulation was done with KLH, a different protein (S3A–S3D Fig). In most cases, the changes in cytokine productions in response to different stimulators were significant except for the cases when ApoB, C5aR and Cpn peptides were used as stimulators for the production of IL-10 in GST-Den-immunized mice, and PAR-1 peptide in RPHC-immunized mice.

The proportion of IL-4 (Th2-related), IL-17A (Th17 related) and IL-2 (Th1-related) expressing CD4^+^ spleen cells from mice immunized with these three constructs was significantly lower (*P*<0.001 for IL-4^+^ and *P*≤0.001 for IL-17A^+^, respectively), showing 2.74 ± 0.11% (AHHC), 2.85 ± 0.12% (RHHC), and 2.87 ± 0.02% (RPHC) for IL-4^+^ when compared to 8.44 ± 0.21% (control) (Fig 4M and 4N); 2.0 ± 0.1% (AHHC), 1.7 ± 0.1% (RHHC), and 0.9 ± 0.1% (RPHC) for IL-17A^+^ when compared to 4.1 ± 0.3% (control) (Fig 4O and 4P). In addition, smaller percentage of CD4^+^IL-17A^+^ expressing spleen cells was observed in RPHC-immunized mice compared to that in either AHHC- or RHHC-immunized mice (*P≤*0.006) (Fig 4O and 4P). Interestingly, higher percentage of CD4^+^IL-17A^+^ expressing spleen cells was observed in RHHC-immunized mice compared to that in RPHC-immunized mice (*P* = 0.021) (Fig 4P). Furthermore, significantly lower percentage of CD4^+^IL-2^+^ expressing spleen cells was observed in three construct-immunized mice when compared to that in control (*P<*0.001; Fig 4Q and 4R). Additionally smaller percentage of CD4^+^IL-2^+^ expressing spleen cells was observed in RPHC-immunized mice compared to that in either AHHC- or RHHC-immunized mice (*P* = 0.019~0.007).

### 5. Evaluation of antigen-induced specific Treg cell function

**Fig 5:**
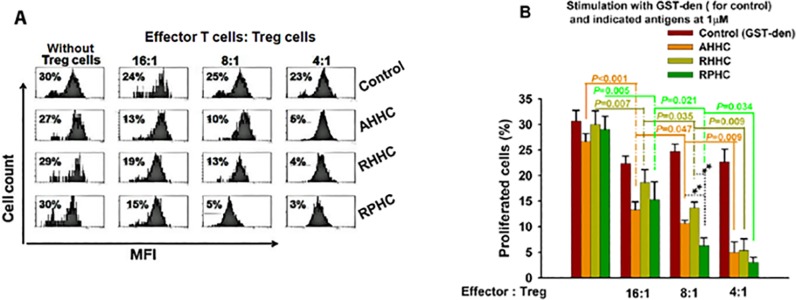
Investigation of antigen-specific regulatory function in antigen-immunized mice. Inhibition of CD4^+^CD25^-^ effector T cell proliferation by CD4^+^CD25^+^ regulatory T-cells isolated from the spleen of control (GST-Den–immunized) and construct-immunized mice when AHHC, RHHC and RPHC were used as antigens. (A) Quantitative analyses of proliferation of CD4^+^CD25^-^ effector T cells in the presence of Treg cell by flow cytometry is shown. (B) Proliferation of effector cells isolated from immunized mice alone is indicated in the leftmost bar of each group. Addition of Treg cells to T effector cells at different ratios was also shown. Data are given as mean of 3 analyses ± SEM.

To assess whether functional Treg cells were induced by immunization, we co-cultured antigen-specific Treg cells (CD4^+^CD25^+^ T cells) with CD4^+^ effector T-cells (CD4^+^CD25^-^ T cells). Proliferation of effector T-cells from control mice immunized with GST-Den in response to stimulation with GST-Den at 1μM did not show suppression in the presence of Treg cells from GST-Den-immunized mice (Fig 5A and 5B). In contrast, proliferation of effector T cells from sampling mice immunized with AHHC, RHHC and RPHC in response to stimulation with related antigen respectively was inhibited (Fig 5A and 5B), when CD4^+^CD25^-^ effector T cells were co-cultured with CD4^+^CD25^+^ Treg cells isolated from these mice. The differences were significant when Treg cells were added to the effector cells at the ratios between 4:1~16:1 (P<0.05~<0.001) compared with that without the addition of Treg cells.

### 6. Evaluation of expression of smooth muscle alpha actins, VCAM1, MMP9 and specific antigens ApoB and HSP60 in the lesions

**Fig 6:**
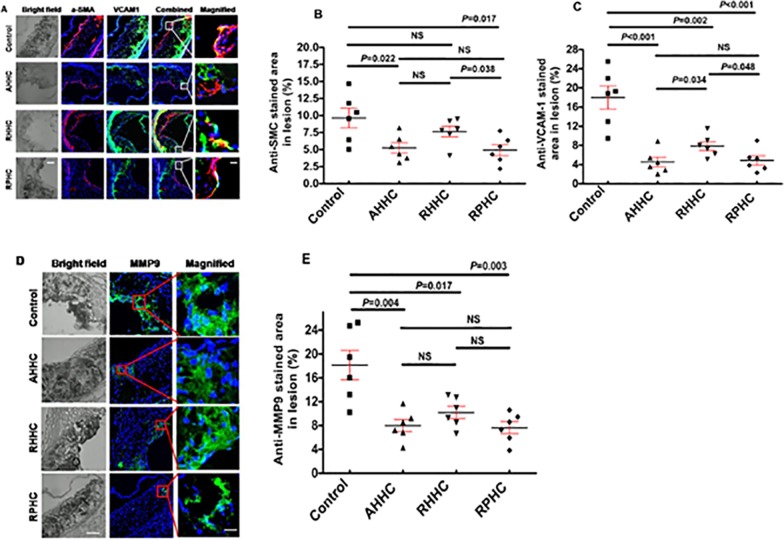
Evaluation of expression of smooth muscle alpha actins, vascular cell adhesion molecule (VCAM)1 and matrix metalloproteinase 9 (MMP9) in lesion site. (A) Photomicrographs showing IHC staining for smooth muscle alpha actin (red), vascular cell adhesion molecule VCAM1(green) (scale bar: 100 μm and 12.5μm for magnified ones). (B) anti-SMC stained area. (C) Scatter plot showing means of anti-VCAM-1 stained area (N = 6). (D) Photomicrographs showing IHC staining for MMP9 (green) (scale bar: 100 μm and 12.5μm for magnified ones). (E) Scatter plot showing means of anti-MMP9 stained area (N = 6).

To assess whether immunization with our constructs influences vascular SMC behavior and vascular remodeling, we analysed the SMC content of lesions and expression of VCAM1 and MMP9 at lesion sites by IHC analyses. Anti-SMC stained area was significantly smaller in the plaques of mice immunized with AHHC and RPHC, showing 5.2 ± 0.7% and 4.9 ± 0.8%, respectively, but it was not reduced significantly in mice immunized with RHHC when compared with that in controls immunized with GST-Den (Fig 6A and 6B). In addition, expression of VCAM1 was significantly down-regulated, showing 4.5 ± 0.9%, 7.8 ± 0.9%, and 4.8 ± 0.9%, for AHHC, RHHC and RPHC, respectively, compared with that in controls immunized with dendroaspin (18.0 ± 2.3%) (Fig 6A and 6C). A significantly increased effect was found in RHHC-immunized mice compared to that in AHHC- or RPHC-immunized mice (Fig 6A and 6C). A similar trend was observed for MMP9 expression in mice immunized with all three constructs 7.9 ± 1.0%, 10.1 ± 1.0%, and 7.6 ± 1.0% stained areas were shown in AHHC-, RHHC- and RPHC-immunized mice, respectively, and 18.1 ± 2.5% in control mice immunized with GST-Den (Fig 6D and 6E), except for that there is no significant difference obtained in this respect between either AHHC- or RPHC-immunized mice and RHHC-immunized mice (Fig 6D and 6E). Interestingly, the injection of recombinant construct containing human ApoB and HSP60 peptides did not affect the expression of their counterparts (ApoB and HSP60) as little difference of mouse ApoB (S4A and S4B Fig) and mouse HSP60 protein (S4C and S4D Fig) antigens was detected at the lesion sites between sampling and control mice.

### 7. Evaluation of monocyte differentiation into macrophages in PBMC from C57BL/6 background naive mice in response to treatment with recombinant constructs; effect of construct-specific immune sera on the differentiation

**Fig 7:**
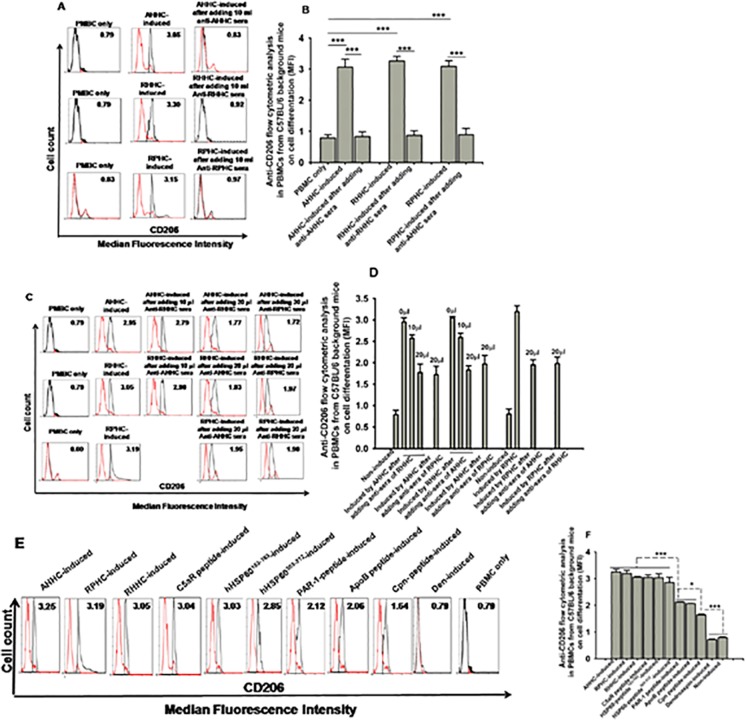
Evaluation of monocyte differentiation into macrophages. (A and B) PBMCs differentiation stimulated by recombinant constructs (1 μg/ml) as assessed by analyses of CD206 expression by flow cytometry and inhibition of differentiation in the presence of respective construct-induced antibodies. (C and D) Inhibition of differentiation by respective and other construct induced antibodies. (E and F) Individual peptide as a stimulator (1 μg/ml) of monocyte differentiation as analysed by CD206 expression. Data are expressed as average values of 3 analyses.

In vitro, monocytes can differentiate into macrophage (or subsets) upon stimulation with macrophage-colony stimulating factor (M-CSF) [21] or atherogenic antigens [17]. To assess whether AHHC converted into RHHC with a single domain substitution could maintain the same effect on stimulation of monocyte (from naive mice C57BL/6 with same background), PBMCs were stimulated with either RHHC or AHHC. After 3 days, the expression of cell surface marker CD206 (mannose receptor, a macrophage marker) was assessed. Both RHHC- and AHHC-induced monocyte differentiation into macrophages (based on the cell number changes) when compared with non-stimulated cells (Fig 7A and 7B). In addition, PBMC differentiation induced by these two constructs was abolished by pre-incubation of the cells with antiserum from mice immunized with these two antigens. Interestingly, the inhibition can be achieved by pre-incubation of the cells with antiserum from each other (Fig 7A–7D). Observation of differentiations with individual epitope or domain as a stimulator showed different ratio of differentiation (Fig 7E and 7F).

### 8. Evaluation of the contents of Toll-like receptor 4(TLR4) and myeloid differentiation factor 88 (MyD88) which are involved in the TLR4 signal pathway related to atherosclerosis at the lesion sites

**Fig 8:**
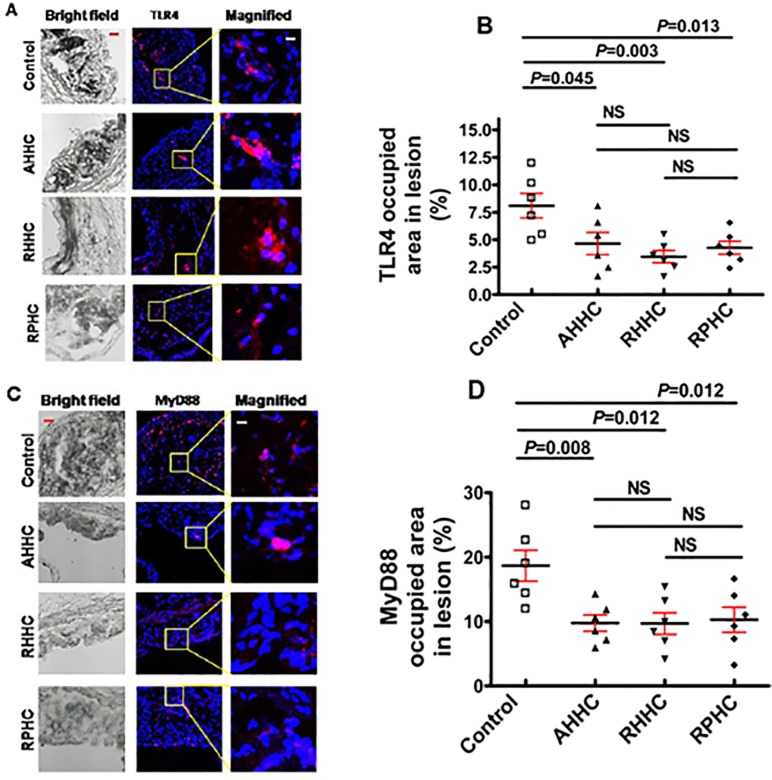
Evaluation of the content of TLR4 and MyD88 contents at the lesion sites. (A) Photomicrographs showing IHC staining for TLR4 (red) (scale bar: 100 μm and 12.5μm for magnified ones). (B) Scatter plot showing means of anti-TLR4-stained area (N = 6). (C) Photomicrographs showing IHC staining for MyD88 (red) (scale bar: 100 μm and 12.5μm for magnified ones). (D) Scatter plot showing means of anti-MyD88-stained area (N = 6).

We examined the impact of the treatment with these recombinant constructs on TLR4 and MyD88 contents in lesions. The reduction of atherosclerosis in mice treated with these constructs was associated with a decrease in both TLR4 and MyD88 contents. Anti-TLR4 stained area was significantly smaller in the plaques of mice immunized with AHHC, RHHC and RPHC, showing 4.6 ± 1.0%, 3.4 ± 0.5% and 4.2 ± 0.6%, respectively, compared with that in controls immunized with dendroaspin (8.1 ± 1.1%) (Fig 8A and 8B). Similarly, anti-MyD88 stained area was significantly smaller in the lesions of mice immunized with these three constructs, showing 9.7 ± 1.2%, 9.6 ± 1.6% and 10.2 ± 1.9%, respectively, compared with that in controls (18.6 ± 2.4%) (Fig 8C and 8D). Additionally, overlapping anti-CD11c and anti-TLR4 stained area showed significantly smaller in the lesions of mice immunized with all three constructs, showing 3.4 ± 0.6%, 2.9 ± 0.7% and 2.7 ± 0.8%, respectively, compared with that in controls (6.6 ± 0.8%) (S5A and S5B Fig).

## Discussion

We have reported the atherosclerotic lesion reducing effect of immunization with a recombinant construct AHHC containing epitopes derived from ApoB, hHSP60 and Cpn [16] and that the C5aR N-terminal peptide has a similar effect on lesion reduction [17]. In this study, we have investigated whether the lesion reducing effect can be increased by using a construct either with a single peptide substitution in AHHC→ RHHC or with double substitutions in AHHC→ RPHC in *B6;129S-Ldlr* ^*tm1Her*^ *Apob* ^*tm2Sgy*^ */J* mice.

PAR-1 peptide was selected for antigen component as immunization with this peptide resulted in reduced lesion formation in a pilot experiment (Xia et al unpublished data). *B6;129S-Ldlr* ^*tm1Her*^ *Apob* ^*tm2Sgy*^ */J* mouse strain was used because these mice show a high degree of atherosclerosis after being fed a high-fat diet [22].

Our results suggest that immunization with all three recombinant constructs induces an antigen-specific antibody response except for PAR-1 and hHSP60 peptide-1. The induced immune response is associated with an anti-atherogenic effect, detected as a significant reduction in the size of the atheromatous lesion area both in aorta sinus and descending aortas with a rate in the following order: RPHC≥ RHHC >AHHC.

Immunization with RHHC in 10-week, high-fat diet (HFD)-based *B6;129S-Ldlr* ^*tm1Her*^ *Apob* ^*tm2Sgy*^ */J* mouse model led to a high antibody response against C5aR peptide. Low level of antibody response against hHSP60^153-163^ and increased antibody responses either against hHSP60^303-312^ or Cpn peptide in RHHC were observed. In RPHC-immunized mice, low IgG responses against PAR-1 peptide and hHSP60 peptide-1, but high antibody responses against C5aR peptide as well as Cpn peptide were achieved. The peptides (e.g. hHSP60^153-163^ or PAR-1 peptide) in either RHHC or RPHC failed to induce IgG response which may be due to steric hindrance or these peptides were not exposed on the surface of the molecule. However, this may not constitute an impact on lesion reduction as according to our previous results AH (containing both ApoB and hHSP60^153-163^ peptides) construct-immunized mice showed higher lesion reduction than either ApoB peptide- or hHSP60^153-163^ peptide-immunized mice [16]. The results from our study also show that immunization induced an IgG1 isotype specific to most antigen peptides when an individual peptide was used as an ELISA antigen. The results of total IgG showed immune response persisting at week 12 (detectable at 100-fold dilutions) in spite of slightly lower OD values at week12 than that at week 2. However, these peptides could induce only a weak peptide-specific IgG2c-isotype response based on the OD values, suggesting that vaccination with these peptides acts predominantly through the Th2 pathway.

Cellular infiltration into atherosclerotic lesions results in increased levels of macrophages, activated CD4^+^ T cells, and dendritic cells (markers of early lesion formation) [23]. We observed that due to immunization with all three constructs, lower numbers of macrophages, and CD11c^+^ cells, and higher number of Treg cells was detectable in the atherosclerotic lesions than in those of control mice and also these numbers showed similar trend when RHHC-immunized mice were compared with AHHC-immunized mice and when RPHC-immunized mice were compared with RHHC-immunized mice.

In agreement with the results of the present study, Cybulsky et al reported that cells bearing markers of dendritic cells (DCs), including CD11c, take up residence just beneath the endothelium in the lesser curvature of aorta in mice [24]. Moreover, the results of our study show that CD68^+^CD11c^+^ cells are the main macrophage subset in lesions, but their functions, and whether their function is affected by the constructs, need to be further explored [20]. Vaccination using mature DCs pulsed with oxidized low-density lipoprotein (oxLDL), or oxLDL-specific T cells with a lowered Th1 response increased the levels of oxLDL-specific antibodies and reduced the lesion size [25]. Therefore, it will be interesting in future studies to determine whether vaccination using mature DCs pulsed with these recombinant constructs could induce specific T cells and could reduce lesion size.

TNF-α and IFN-γ are crucially involved in the progression of atherosclerosis [26–28]. On the basis of the cytokine profiles from either plasma or supernatant of splenocytes in immunized mice, TNF-α and IFN-γ secretion may well be linked to the ability of the immune animals to release these cytokines from preprimed cells, leading to a decrease in the need to synthesize significant amounts of new TNF-α and IFN-γ. Our results show that immunization with RPHC, RHHC and AHHC promoted a major shift away from proinflammatory cytokines (TNF-α and IFN-γ) towards anti-inflammatory cytokines (IL-10 and TGF-β), which is evident not only in the plaque but also systemically at remote sites (splenocytes). These results strongly suggest that an anti-inflammatory response is responsible for the observed reduction in plaque size, they do not discriminate the reductions attributed to either of the anti-inflammatory cytokines, as both IL-10 and TGF-β, the two major cytokine products of Treg, have been implicated in playing a role in atherosclerosis [29,30]. Our results also showed these antigens have antigenic effect which elicited increased atheroprotective cytokine IL-10 (Th2 cytokine) and decreased proinflammatory cytokine IFN-γ (Th1 cytokine) in spleen cell of antigen-immunized mice. Further evidence was obtained from CD4^+^ T cell expressing cytokines, IL-4, IL-17A and IL-2. IL-4, a major cytokine directing differentiation of Th0 cells to Th2 cells [31], is the signature cytokine of Th2 lymphocytes and expressed by T cells in atheroma of severely hypercholesterolemic ApoE^−/−^ mice [32]. IL-4 has been shown to influence cell adhesion by causing an increase in expression of VCAM1 by endothelial [33] and of vascular smooth muscle cells [34], whereas IL-17A, a predominant IL-17 family member, have recently been detected within human and mouse atherosclerotic vessels [35]. Elevated plasma IL-17 was found in patients with coronary artery disease (CAD) in comparison with healthy controls [36]. In addition, expression of IL-17A in human atherosclerotic lesions is associated with increased inflammation and plaque vulnerability [37]. Furthermore, the presence of both IL-17A and IFN-γ in clinical specimens of coronary atherosclerosis and the presence of IL-17A/IFN-γ double producer T cells within coronary plaques were reported [36]. In agreement with the data on the IL-4 and IL-17A, our results showed that the number of CD4^+^ T cell expressing IL4 and IL-17A in mice immunized with our three constructs was significantly reduced. In addition, the lowest percentage of the IL-17A expressing CD4^+^ T cell population was achieved by immunization with RPHC construct as antigen, whereas lower percentage of CD4^+^ T cell expressing IL-17A was also observed in RHHC-immunized mice than in AHHC-immunized mice. Furthermore, our results showed that the number of CD4^+^ T cell expressing IL-2 (Th1 cell-related) in mice immunized with our three constructs was significantly decreased. IL-2, a pro-inflammatory T cell cytokine, is a cytokine mainly produced by activated T cells [38]. Our results suggest that IL-2 may promote atherosclerosis.

Next we investigated whether Tregs played a role in reducing atherosclerotic lesion formation by immunization with RPHC and RHHC as well, in case of AHHC it is, at least in part, due to increased Treg cell concentration in immunized mice, either locally (in the lesions) or in remote organs (splenocytes): we observed that concentrations of CD4^+^ Treg cells were significantly higher in mice immunized with recombinant constructs than of those either locally (in lesion site of aorta) or in remote organs (splenocytes) in control mice immunized with GST-Dendroaspin. In addition, Foxp3^+^ population in RPHC-immunized mice was significantly higher than that in AHHC-immunized mice, suggesting functional and structural differences between these two antigens that may contribute to the increased Foxp3^+^ population. We also demonstrated that the atheroprotective effect paralleled an induction of functional Treg cells that suppressed antigen-specific proliferation of effector T cells, thus suggesting that Tregs have an active role in the control of the atherosclerotic process. Treg cells are characterized by the expression of Foxp3, which has a crucial role in their suppression function [35].

Clinical data showed that Th17/Treg functional imbalance exists in patients with acute coronary syndrome (ACS), a significant decrease in Treg number, Treg-related cytokines (IL-10 and TGF-β1), and Foxp3 levels, suggesting a potential role for Th17/Treg imbalance in plaque destabilization and the onset of ACS [39]. It appears that modulation of atherosclerosis-related autoimmunity by antigen-specific activation of Tregs represents a novel approach for the treatment of atherosclerosis.

It is known that expression of smooth muscle alpha actin is altered in pathological conditions of atherosclerosis and the existence of alpha-smooth muscle actin-positive endothelial cells in adult aortic endothelium is associated with progression of atherosclerosis [40]. In addition, the atherosclerotic vessel wall contains a range of adhesion molecules [41] and inflammatory cytokines [42], which may permit attachment and subsequent differentiation of marrow derived cells into smooth muscle cells within plaque [43]. Furthermore, several matrix metalloproteinases have been reported to play roles in atherosclerosis such as MMP-9 [44]. The lesion development is initiated by infiltrating macrophages that mainly produce MMP-9 as well as MMP2 [45]. Our results showed that mice immunized with all three antigens have the lower expression of alpha-SMCs, VCAM1 and MMP9 when compared to the control animals.

Monocytes play important roles in the initiation, progression, and complications of atherosclerosis. Their differentiation to macrophages can be modulated by factors present within the microenvironment of the artery wall, such as oxidized lipids, TLR ligands, cytokines, and chemokines [21]. It should be noted that macrophages can be differentiated into various macrophage subsets with different cell surface markers; further discrimination of macrophage subsets is planned in our future studies. Our in vitro study showed that these constructs can be used as stimulators of monocyte to macrophage differentiation. The antibodies against these constructs can be served as inhibitors in this respect, suggesting antibody elicited by immunization may have similar property to reduce monocyte differentiation in vivo, as ApoB, HSP60, C5aR (from which the peptide epitope derived) were detected in the atherosclerotic lesion sites in our present and previous studies [17] which may serve as endogenous ligands.

Additionally, macrophages serve as the major immune cells in the lesion and they express pattern-recognition receptors, including Toll-like receptors (TLRs) that connect the innate and adaptive immune response during atherosclerosis. The mechanisms involved in signaling pathways, which link TLR4 (also serving as a Th1 marker) to the proinflammatory transcription factor nuclear factor κB (NF-κB), occur through MyD88, a general adaptor protein downstream of TLRs (except for TLR3) and the receptors of the IL-1R family [46,47]. MyD88 signaling causes defects in pro-inflammatory cytokine production and innate immune cell activation, permitting the development of a high pathogen burden [48]. In agreement with the report by Subramanian et al. [49], our results show that both TLR4 and MyD88 contents as well as common occupied lesion area by TLR4 and CD11c were significantly reduced in mice immunized with recombinant constructs, indicating that TLR4/MyD88 pathway was down-regulated in these antigen-immunized mice, however, the pathways that link TLRs to pro-atherogenic effectors in immune cells remain under investigation.

Collectively, the results showed that all three constructs are potent antigenic constructs and these constructs can be modulated towards more effective inhibition of atherosclerotic plaque formation via reducing proinflammatory cells, cytokines and increasing anti-inflammatory cells, cytokines and the proportion of Tregs as statistically shown in S1 Table. The effect of change of immune parameters in the plaques, cell-to-cell interaction (Treg cell and T effector cell; Treg cell and monocyte/macrophage) and immune cross-reactivity between paired epitopes (A, R and P) may contribute to favorable plaque phenotype and increased lesion reduction as shown in RHHC and RPHC. The immune response induced against the involved peptide antigens reduces the plaques in mice. It is acceptable that ApoB and HSPs are self antigens which might induce immune response causing damage to the vessel wall and cause inflammation. In this case, these antigens induced regulatory T cells which can be atheroprotective. In the case of PAR-1 and C5aR, their antibodies may be able to prevent activations of atherogenic pathways. Further study on knockout mice with these receptors are needed.

The study highlights application of antigen-specific immunomodulation in which Treg-mediated suppression of atherosclerosis requires TLR4/MyD88 signaling and this immunomodulation permits selective inactivation of auto-reactive T cells without interfering with normal immune function [46] as our results have shown the similar profiles of autoantigen ApoB and HSP60 at the lesion sites in both control and sampling animals. Indeed sequence alignment by using“SIM” software shows 69.7% and 97.6% identity for ApoB and HSP60, respectively between mouse and human (data not shown). Atheroprotection by immunization with these construct is likely to be mediated by both a humoral and a cell-mediated regulatory response to the antigen. Our findings have suggested that developing a protein-based vaccine using a dendroaspin scaffold with rearrangement of different peptide sequence(s) may offer attractive opportunities for the development of multivalent vaccines against atherosclerosis.

## Supporting Information

S1 Fig Schematic representations of dendroaspin structure and constructs. **A.** Schematic representation of the backbone of dendroaspin structure [1]. **B.** Schematic representation of alignment of constructs in dendroaspin scaffold.
(TIF)

S2 Fig Detection and quantitation of the lesion areas in the aorta of *B6;129S-Ldlr* ^*tm1Her*^ *Apob* ^*tm2Sgy*^ */J* mice fed on a high-fat diet at week 9 after immunization with constructs versus controls (GST-Den), GST and alum. **A.** Photomicrograph of lesions observed in atherosclerotic aortas as analyzed with elastin/van Gieson staining (N = 6–8 mice). **B.** Scatter plot showing mean of lesion area in the aortic sinus of mice (N = 6-8mice).
(TIF)

S3 Fig Assessment of ApoB and HSP60 in the lesions of *B6;129S-Ldlr* ^*tm1Her*^ *Apob* ^*tm2Sgy*^ */J* mice fed a high-fat diet after immunization with constructs AHHC, RHHC, and RPHC. **A.** Representative photomicrographs showing immunohistochemical staining of the aortic root showed anti-ApoB antibody stained areas (red) in the lesion. **B.** Quantitative analysis of ApoB content in lesions. **C.** Representative photomicrographs showing immunohistochemical staining of the aortic root showed anti-HSP60 antibody stained areas (red) in the lesion. **D.** Quantitative analysis of HSP60 content in lesions. Scale bar: 150 μm (unenlarged) and 25 μm (enlarged). N = 6 mice. NS: not significant.
(TIF)

S4 Fig Measurement of cytokines in the supernatant of spleen from immunized mice stimulated by either GST-Den or constructs. **A.** IFN-γ from GST-Den-immunized mice. **B.** IFN-γ from construct-immunized mice). **C.** IL-10 from GST-Den-immunized mice. **D.** IL-10 from construct-immunized mice). Splenocytes were cultured in RPMI 1640 with 10% fetal calf serum and induced with1 μg/ml antigen (GST-Den, GST-AHHC, GST-RHHC, GST-RPHC, ApoB100 peptide, C5aR peptide, PAR-1 peptide, respectively) for 48 hour. Then IL-10 and IFN-γ in supernatant of cultured cell were measured with DuoSet mouse IL-10 kit and mouse IFN-γ Quantikine immunoassay kit (R&D system, Minneapolis) according to manufactory’s protocol.
(TIF)

S5 Fig Detection and quantitation of TLR4 overlapped with CD11c in lesion area. **A.** Representative photomicrographs showing immunohistochemical staining of the aortic root showed anti-TLR4 antibody stained areas (red) overlapped with anti-CD11c antibody stained area (green) in the lesion (overlapped area is in yellow). **B.** Measurement of combined (TLR4 and CD11c) area occupied in lesion (%). Scale bar: 141 μm (unenlarged) and 26 μm (enlarged).
(TIF)

S1 Table Statistical analysis of the effects of immunization with the constructs. (DOCX)

S1 Text Materials and methods. (DOCX)
