# Supplementary figures and images for: Modulation of Recombinant Antigenic Constructs Containing Multi-Epitopes towards Effective Reduction of Atherosclerotic Lesion in B6;129S-Ldlrtm1HerApobtm2Sgy/J Mice
Source: PLoS One. 2015 Apr 1;10(4):e0123393. doi: 10.1371/journal.pone.0123393 (PMC4382319; doi:10.1371/journal.pone.0123393)

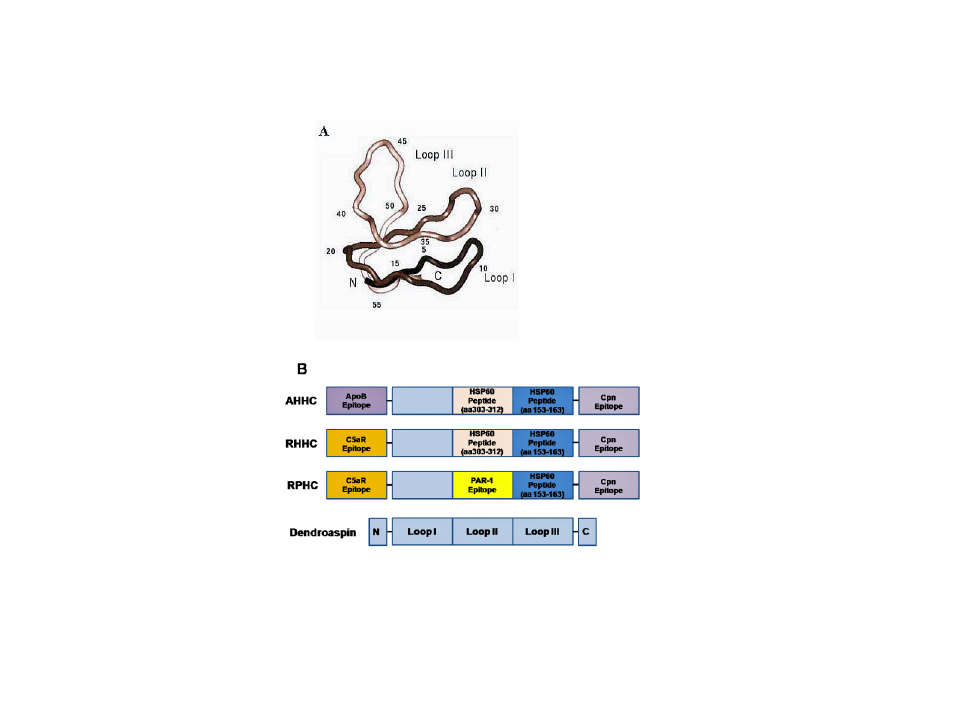

Supplement: S1 Fig — A. Schematic representation of the backbone of dendroaspin structure [1]. B. Schematic representation of alignment of constructs in dendroaspin scaffold. (TIF) [file pone.0123393.s001.tif]

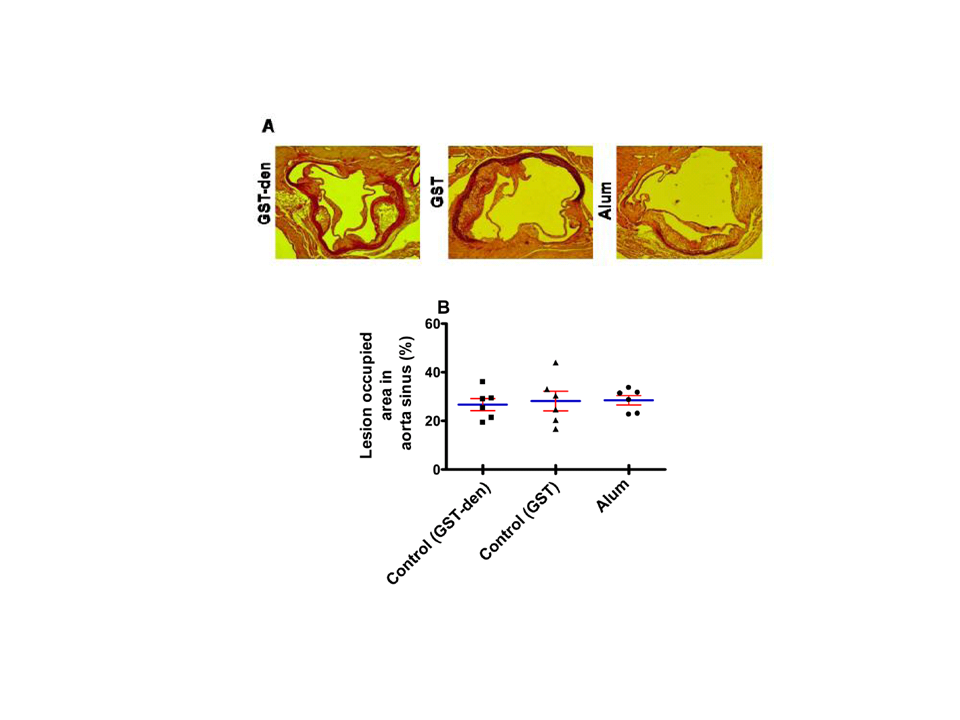

Supplement: S2 Fig — A. Photomicrograph of lesions observed in atherosclerotic aortas as analyzed with elastin/van Gieson staining (N = 6–8 mice). B. Scatter plot showing mean of lesion area in the aortic sinus of mice (N = 6-8mice). (TIF) [file pone.0123393.s002.tif]

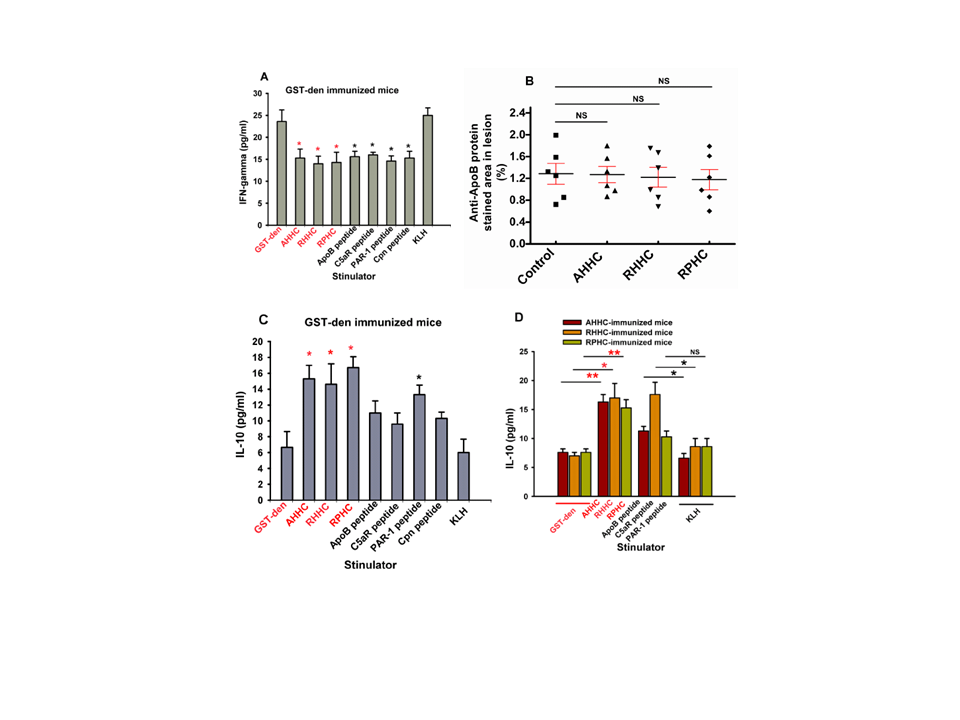

Supplement: S3 Fig — A. Representative photomicrographs showing immunohistochemical staining of the aortic root showed anti-ApoB antibody stained areas (red) in the lesion. B. Quantitative analysis of ApoB content in lesions. C. Representative photomicrographs showing immunohistochemical staining of the aortic root showed anti-HSP60 antibody stained areas (red) in the lesion. D. Quantitative analysis of HSP60 content in lesions. Scale bar: 150 μm (unenlarged) and 25 μm (enlarged). N = 6 mice. NS: not significant. (TIF) [file pone.0123393.s003.tif]

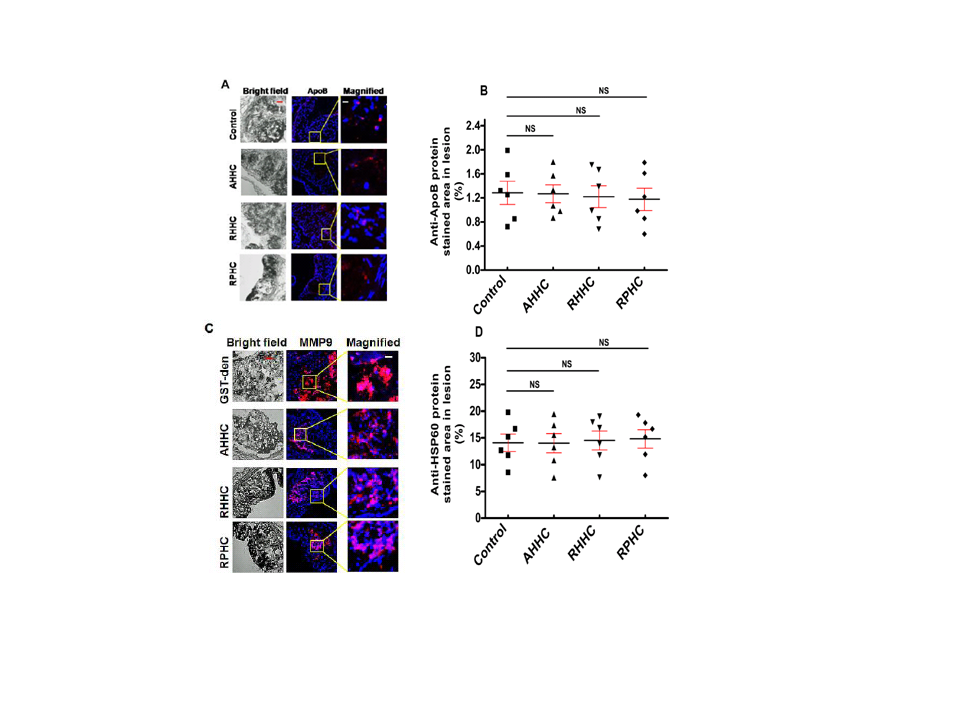

Supplement: S4 Fig — A. IFN-γ from GST-Den-immunized mice. B. IFN-γ from construct-immunized mice). C. IL-10 from GST-Den-immunized mice. D. IL-10 from construct-immunized mice). Splenocytes were cultured in RPMI 1640 with 10% fetal calf serum and induced with1 μg/ml antigen (GST-Den, GST-AHHC, GST-RHHC, GST-RPHC, ApoB100 peptide, C5aR peptide, PAR-1 peptide, respectively) for 48 hour. Then IL-10 and IFN-γ in supernatant of cultured cell were measured with DuoSet mouse IL-10 kit and mouse IFN-γ Quantikine immunoassay kit (R&D system, Minneapolis) according to manufactory’s protocol. (TIF) [file pone.0123393.s004.tif]

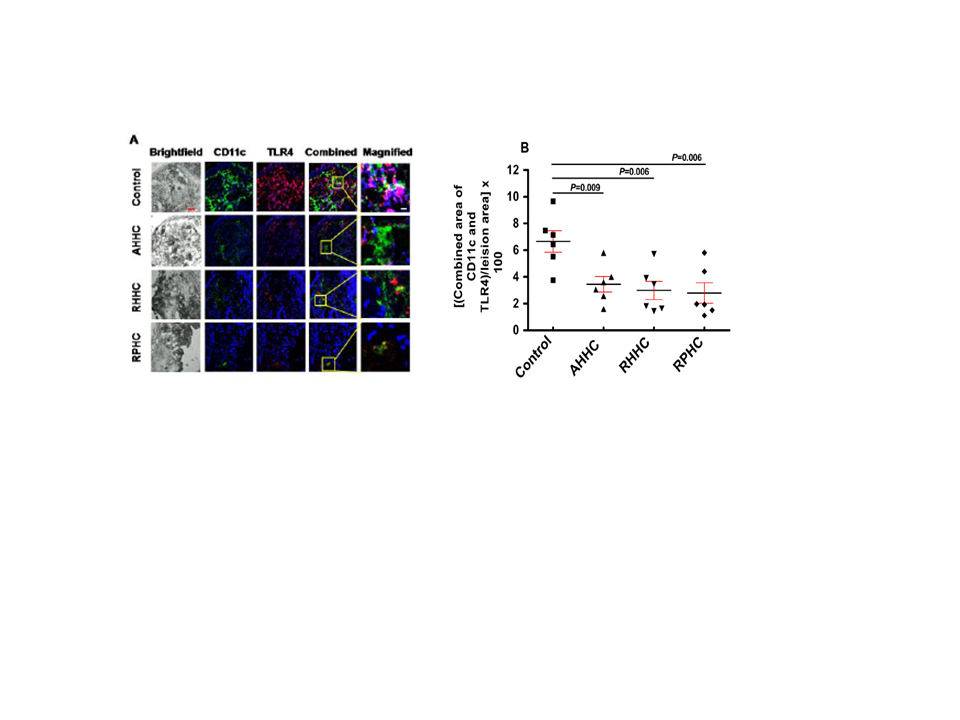

Supplement: S5 Fig — A. Representative photomicrographs showing immunohistochemical staining of the aortic root showed anti-TLR4 antibody stained areas (red) overlapped with anti-CD11c antibody stained area (green) in the lesion (overlapped area is in yellow). B. Measurement of combined (TLR4 and CD11c) area occupied in lesion (%). Scale bar: 141 μm (unenlarged) and 26 μm (enlarged). (TIF) [file pone.0123393.s005.tif]
