# Supplementary material for: Modulation of Recombinant Antigenic Constructs Containing Multi-Epitopes towards Effective Reduction of Atherosclerotic Lesion in B6;129S-Ldlrtm1HerApobtm2Sgy/J Mice
Source: PLoS One. 2015 Apr 1;10(4):e0123393. doi: 10.1371/journal.pone.0123393 (PMC4382319; doi:10.1371/journal.pone.0123393)
Supplement: S1 Table — (DOCX) [file pone.0123393.s006.docx]

**S1Table. Statistical analysis of the effects of immunization with the constructs**

|  | **AHHC**  **(A)** | **RHHC**  **(B)** | **RPHC**  **(C)** | **Statistical analysis** |
| --- | --- | --- | --- | --- |
| **Lesion occupied areas in aortas (μm)^2^** | **31071** | **24123** | **21386** | **B vs A, *P*<0.01; C vs A, *P*<0.01** |
| **Lesion reduction in aortas (%)** | **55.9** | **66.4** | **68.9** | **B vsA, *P*<0.01; C vs A, *P*<0.01** |
| **Lesion occupied areas in descending aortas (%)** | **8.4** | **6.6** | **6.4** | **B vs A, *P*<0.05; C vs A, *P*<0.01** |
| **Lesion reduction in descending aortas (%)** | **57.3** | **66.1** | **67.3** | **NS** |
| **Collagen content in lesions (%)** | **18.6** | **19.4** | **24.4** | **C vs A, *P*<0.01;C vs B, *P*<0.01** |
| **Macrophage-occupied area in lesion (%)** | **14.9** | **13.6** | **10.3** | **C vs A, *P*<0.05** |
| **Dendritic cell-occupied area (%)** | **13.3** | **10.5** | **7.9** | **C vs A, *P*<0.05** |
| **CD4^+^ T cell expressing Foxp3 (%) in lesion** | **8.2** | **9.4** | **9.9** | **NS** |
| **CD4^+^ T cell expressing Foxp3 (%) in splenocytes** | **13.3** | **15.3** | **18.0** | **C vs A, *P*<0.01** |
| **CD4^+^ expressing IL-10 in lesions** | **4.8** | **5.2** | **5.9** | **NS** |
| **TNF-α-occupied areas in lesions** | **25.7** | **15.2** | **15.6** | **B vs A, *P*<0.01;C vs A, *P*<0.05** |
| **IL-10 in plasma (pg/ml)** | **10.0** | **12.3** | **15.9** | **C vs A, *P*<0.01** |
| **TGF-β in plasma (pg/ml)** | **23.0** | **24.3** | **27.2** | **C vs A, *P*<0.01;C vs B, *P*<0.05** |
| **TNF-α in plasma (pg/ml)** | **209** | **200** | **180** | **C vs A, *P*<0.001;C vs B, *P*<0.01** |
| **IFN-γ in plasma (pg/ml)** | **20.8** | **19.3** | **18** | **C vs A, *P*<0.01** |
| **IL-10 in supernatants of splenocytes (pg/ml) stimulated with 10 µg/ml ConA** | **34** | **38** | **40** | **NS** |
| **TGF-β in supernatants of splenocytes (pg/ml) stimulated with 10 µg/ml ConA** | **35** | **39** | **43** | **NS** |
| **TNF-α in supernatants of splenocytes (pg/ml) stimulated with 10 µg/ml ConA** | **217** | **209** | **195** | **NS** |
| **IFN-γ in supernatants of splenocytes (pg/ml) stimulated with 10 µg/ml ConA** | **45** | **44** | **42** | **NS** |
| **IL-4^+^ expressing CD4^+^ T cells (%)** | **2.7** | **2.8** | **2.8** | **NS** |
| **IL-17A^+^ expressing CD4^+^ T cells (%)** | **2** | **1.7** | **0.9** | **C vs A, *P*<0.001; C vs B, *P*<0.01** |
| **IL-2^+^ expressing CD4^+^ T cells (%)** | **4** | **3** | **3** | **C vs A, *P*<0.01; C vs B, *P*<0.05** |
| **Smooth muscle cell expression in lesion (%)** | **5.2** | **7.6** | **4.9** | **C vs A, *P*<0.05** |
| **VCAM1 (%)** | **4.5** | **7.8** | **4.8** | **B vs A, *P*<0.05; C vs B, *P*<0.05** |
| **MMP9 (%)** | **7.9** | **10.1** | **7.6** | **NS** |
| **TLR4 (%)** | **4.6** | **3.4** | **4.2** | **NS** |
| **MyD88 (%)** | **9.7** | **9.6** | **10.2** | **NS** |

**NS: denotes not significant**
