# Supplementary material for: Modulation of Recombinant Antigenic Constructs Containing Multi-Epitopes towards Effective Reduction of Atherosclerotic Lesion in B6;129S-Ldlrtm1HerApobtm2Sgy/J Mice
Source: PLoS One. 2015 Apr 1;10(4):e0123393. doi: 10.1371/journal.pone.0123393 (PMC4382319; doi:10.1371/journal.pone.0123393)
Supplement: S1 Text — (DOCX) [file pone.0123393.s007.docx]

**MATERIALS AND METHODS**

**Generation of recombinant constructs AHHC, RHHC and RPHC.**

Schematic representation of the backbone of the dendroaspin structure is shown in S1A Fig. [1].

The construct AHHC contains an epitope derived from human ApoB100 (peptide sequence: I**^688^**EIGLEGKGFEPTLEALFGK**^707^**, numbered including signal peptide) at the N-terminal of dendroaspin with a poly-glycine linker between ApoB peptide and dendroaspin; an epitope of hHSP60 (A**^153^**ELKKQSKPVT**^163^**) in dendroaspin loop III as a replacement of wild-type loop III sequence and an epitope derived from hHSP60 (peptide sequence: P**^303^**GFGDNRKNQ**^312^**) in dendroaspin loop II; and a *Cpn* peptide, a combination of sequences derived from the major outer membrane protein (MOMP) of the *Cpn* (peptide sequence: G**^67^**DYVFDRI**^74^**) and from the outer membrane protein (Omp) 5 of *Cpn* (peptide sequence: Q**^283^**AVANGGAI**^291^**). The construct RHHC was derived from AHHC in which ApoB peptide was replaced by a sequence derived from C5aR (peptide sequence M^1^NSFNYTTPDYGHYDDKDTLDLNTPVDKTSN^31^), similarly RPHC was derived from RHHC in which hHSP60 peptide two was replaced by a sequence from PAR-1 (peptide sequence S^42^FLLRNPNDKYEPF^55^). Schematic representation of constructs is shown in S1BFig.

The genes were synthesized by Genescript, USA Inc under confidential agreement and cloned into a PUC57 vector. A PCR was employed for producing the genes of these three constructs converting a *HindIII* restriction site within PUC57 vector into an *EcoRI* site which permits cloning into pGEX-3X vector. AHHC construct was described previously [2]. For other two constructs, the following primers were used: a forward primer with a *BamHI* cleavage site: 5’‑GAA GGG ATC CAT ATC GAA GGT CGT ATG AAC TCT TTC-3’ and a reverse primer with an *EcoRI* site: 5’-TCA TCA GAA TTC TCAGAT AGC ACC ACC GTT AGC GAC AGC CTG ACC ACC ACC ACC ACC GAT ACG were used, but with different templates. For the PCR reactions, 100 μl of reaction mixture prepared in either *Taq* polymerase reaction buffer (50 mM KCl; 10 mM Tris-HCl, pH 8.8; 1.5 mM MgCl_2_; 0.1 % Triton X-100) or *Vent* polymerase reaction buffer [10 mM KCl; 10 mM (NH_4_)_2_SO_4_; 20 mM Tris-HCl, pH 8.8; 2 mM MgSO_4_; 0.1 % Triton X-100] containing ddNTP (400 μM ), 300 ng of each oligonucleotide primer, 25–100 ng cDNA template and 1.5–2 units of either *Taq* or *Vent* DNA polymerase were placed in a Perkin-Elmer/Cetus Thermal Cycler (Norwalk, CT, USA). After initial denaturation of the DNA at 94^o^C for 2.5 min, a cycle program was set: 40 cycles- 94^o^C for 45 seconds, 70^o^C for 2 minutes and 55^o^C for 2 minutes. After PCR, 10 μl of each reaction mixture was electrophoretized in an agarose gel and visualized by ethidium bromide staining to check the yield. The PCR fragments were digested by *EcoRI* and *BamHI* and gene-cleaned to separate DNA templates from the newly synthesized genes, and cloned into the carboxyl terminus of the glutathione S-transferase (GST) gene in the restricted vector pGEX-3X (Amersham Biosciences UK Limited). The genes were transformed into an *E. coli* DH5α strain.

Dendroaspin (Den) contains four disulphide bridges and has an adaptable protein template that can be manipulated by the substitution and insertion of peptide sequences [3]. hHSP60^153-163^ was inserted into loop III of dendroaspin (11 amino-acid residues) in three constructs. Loop III was selected as a site for substitution since it is solvent-exposed and located away from other loops; consequently, with the exception of construct A, the Arg-Gly-Asp (RGD)-tripeptide thought to be an integrin-binding site and its flanking sequence [4] were substituted by hHSP60^153-163^ in all other constructs. Although the RGD-tripeptide is not substituted in construct A, this tripeptide along with its flanking sequence do not contribute to lesion reduction, as immunization with a wild-type dendroaspin without coupled immunogen was associated with the same grade of lesion as those in non-immunized controls. In addition, we attached a combination of *Cpn* peptide (26 residues) onto the C-terminals of three constructs. This combination of *Cpn* peptide was proved to be immunogenic and showed cross-reactivity with ApoB peptide in our earlier studies studies [2, 5]. Furthermore, we substituted loop II with hHSP60^303-312^ epitope in construct RHHC and AHHC, this modification in the latter construct has been shown to have an additional effect on the immune response in reducing the atherosclerotic lesions. Moreover, we also replaced hHSP60 peptide (sequence: P**^303^**GFGDNRKNQ**^312^** with PAR-1 peptide (sequence S^42^FLLRNPNDKYEPF^55^) in order to determine whether this replacement in RPHC can increase lesion reducing effect. The genes of constructs were cloned into a DNA vector PGEX-3X so that they were expressed as glutathione S-transferase-fusion protein for the purpose of increasing protein yield as well as for affinity purification.

**Transformation of *E. coli* DH5α and BL21**

The pGEX-3X vector with insertions of the genes of three constructs were used to transform 50 μl of *E. coli* DH5α (for preparing plasmid DNA) or BL21 (for protein expression) competent cell line by incubation on ice for 30 minutes followed by a heat shock of 20 s at 37^o^C. Following further 2-minute incubation on ice, the cells were shaken at 37^o^C for 60 minutes in growth medium (1.0 ml) and were then plated. The positive colonies on the LB plate were screened by PCR. Presence of the correct coding sequence of gene was verified by dideoxy chain termination sequencing.

**Protein expression**

Protein expression in *E. coli* was performed as described previously [2]. In brief, bacterial culture conditions were set up as follows; the 2xYT/ampicillin medium (100 μg/ml) was inoculated with an overnight seed culture (1%, v/v), it was shaken at 37^o^C until it reached an OD600 of 0.7. Isopropyl β-D-thiogalactoside (IPTG) was then added to a final concentration of 0.1 mM for induction. The cells were grown for additional 4 hours at a low temperature of 30°C and harvested by centrifugation.

**Affinity and ion exchange chromatography**

GST-fusion constructs were prepared from the sonicated cells of *E. coli* by affinity chromatography using glutathione-Sepharose 4B columns followed by DE50 ion-exchange chromatography. Purified GST-fusion proteins were analyzed by SDS-PAGE for homogeneity. The working concentration of the GST-fusion proteins was determined by a combination of protein estimation with the Micro BCATM-Protein Assay Reagent kit (Pierce) with BSA as an internal standard and protein estimation on SDS-PAGE (BSA as a standard) analyzed by a Bio-Rad gel scanner (GelDoc 2000).

**Animal experiments:**

**Animals:** Male *B6;129S-Ldlr^tm1Her^Apob^tm2Sgy^/J* mice (The Jackson Laboratory) were maintained under standard husbandry conditions at the animal facility of the Department of Medical Microbiology and Immunobiology, University of Szeged and were given food and water ad libitum. The sampling and control groups consisting of 5-6-week-old males in each group were established. The experiment was repeated twice. For sample groups, immunizing antigens used were constructs AHHC, RHHC and RPHC. Mice in control groups were immunized with dendroaspin (Den). For antigen injection, the "repetitive immunization multiple sites strategy" (RIMMS) was adopted [6,7]. Mice were inoculated 5 times at 2-3-day intervals under pentobarbital sodium anesthesia. At each immunization mice received 20 μg protein combined with Aluminum hydroxide adjuvant (0.65 mg/mouse; Alum, Aluminum hydroxide gel from Sigma) in PBS with a final volume of 240 μl. Two weeks after the first antigen injection, sera were collected for ELISA test. Blood was drawn from the heart of 9-10-week old C57BL6 mice under deep pentobarbital sodium anesthesia. Heparin-anti-coagulated blood was separated by using Histopaque-1083 (Sigma) to obtain peripheral blood mononuclear cells (PBMC).

The experiments were approved by the Animal Welfare Committee of the University of Szeged and conform with to the Directive 2010/63/EU of the European Parliament.

**Antibody response measurement**

Blood samples were collected in heparinized capillaries by retro-orbital bleeding under pentobarbital sodium anesthesia at week two, and twelve weeks after the first injection of the antigens to test antibody production. The free ApoB-100 peptide, two different hHSP-60 peptides, C5aR peptide, PAR-1 peptide and Cpn peptide containing an N-terminal cysteine (synthesized by Severn Biotech Ltd, UK) were used in ELISA as antigens. Maleimide activated 96-well plates (Pierce, Thermo Fisher Scientific Inc., USA) were coated with these peptides individually and peptide-specific IgG was measured in the plasma of immunized mice according to the manufacturer’s instructions. Horseradish peroxidase-conjugated α-mouse IgG (Jackson ImmunoResearch Laboratories, Inc. West Grove, PA, USA) was used as secondary antibody. 1:100 dilution of plasma samples was made before assaying for peptide-specific IgG. Peptide-specific IgG1 and IgG2c were also detected in the plasma samples of immunized mice. For IgG1 measument 1:6250 dilution and for IgG2c level 1:50 dilution of plasma samples were used. Horseradish peroxidase (HRP)-conjugated anti-mouse IgG1 (Biosource, San Joe, CA, USA) and HRP-conjugated anti-mouse IgG2c (Abcam, Cambridge, UK) were used as the secondary antibodies.

**Tissue preparation**

Twelve weeks after the first immunization, hearts with proximal aortas were harvested and mounted in OCT or paraffin, for immunohistochemical analyses and lesion measurement, respectively. From the samples frozen in OCT medium (OCT compound, Tissue-Tek, Sakura Finetek, Europe) 5-μm thick sequential sections were taken using a Reichert-Jung Cryocut 1800 (Leica). The paraffin-embedded sections were prepared using a Leica Jung RM2055 microtome.

Spleens, inguinal and axillary lypmph nodes were dissected and one third of spleen of each mouse was embedded in OCT for immunohistochemical analyses, two third of the spleen and lymph nodes were homogenized by pressing through a 70 μm nylon cell strainer and the cells were recovered in RPMI 1640 complete medium supplemented with 10% FCS, 100 U/ml penicillin and 100 μg/ml streptomycin.

**Morphometric analyses and quantitative measurements of atherosclerosis**

Paraffin-embedded sections were cut serially at 8 μm intervals from the aortic sinus and mounted on slides. Prior to staining, sections were de-paraffinized in xylene and rehydrated in graded series of ethanol. For area measurements and morphometric analysis, slides prepared from formalin-fixed sections were stained with hematoxylin and eosin (HE) and elastin/van Gieson (Sigma) for histological evaluation using an Olympus U-ULH optical microscope (Olympus Optical Co. Ltd, Japan). Image-Pro Plus TM software version 4.0 (Media Cybernetics, Silver Spring, USA) was used to trace the external elastic lamina, internal elastic lamina and lumen in the sections of aortic root area to ascertain area of atherosclerotic lesions. The total cross-sectional aortic area (measured along the inner aortic perimeter) and lesion area were measured following which the ratio of total lesion area to total aortic lumen area was calculated and expressed as a percentage of lesion in the cross-section of the aortic root area. Quantitative analysis of collagen content of sections was done by applying Sirius Red coloration. Longitudinally opened descending aortas were evaluated for the extent of atherosclerosis after Oil Red O (ORO) staining.

**Immunohistochemical analyses**

Hearts with proximal aortas and spleens embedded in OCT were sectioned and sections with aortic sinus were fixed in methanol. Consecutive tissue sections were incubated in 0.2% TritonX-100/PBS for 1 hour then blocked with 1% BSA for 1 hour. The samples were stained with either purified hamster anti-mouse CD11c (eBioscience, Ltd., Hatfield, UK) or rat anti-mouse CD4 (BD Biosciences, Oxford, UK), CD68, IL-10 or TNF-α (BioLegend, London, UK) at 4°C overnight, washed in PBS and incubated with goat anti-hamster IgG-TRITC (Sigma, UK) or rabbit anti-rat IgG-FITC. For smooth muscle α-actin detection, rabbit anti-mouse SMAalpha and goat anti-rabbit IgG-TRITC (Abcam, Cambridge, UK) were used. For detection of MMP9, VCAM1(CD106), HSP60, ApoB protein, TLR4 and MyD88 rabbit anti-mouse polyclonal antibodies of these proteins (Abcam, Cambridge, UK) and FITC or TRITC conjugated goat anti-rabbit IgG were used.

All slides were counterstained with mounting medium containing DAPI (Vector Laboratories Inc., USA). Bright-field images were captured, scanned and overlaid using an Axiovert S100 TV immunofluorescence microscope (Zeiss, UK) equipped with Plan-NEOFLUAR objectives and a KTL/CCD-1300/Y/HS camera from Princeton Instruments, USA. Image-Pro Plus TM software version 4.0 was used to determine lesion area. The CD68^+^, CD11c^+^, Foxp3^+^ and CD4^+^ areas within the lesions and CD4^+^ areas in spleen sections were measured with a microscope and averaged in μm^2^. Three sections from each animal (six animals from each group) were investigated.

**Measurement of the levels of CD4^+^ T cell expressing Foxp3, IL-2, IL-4, IL-17A and monocyte differentiation by flow cytometry**

FITC-conjugated rat anti-mouse CD4 mAb (eBiocience Ltd., Hatfield, UK), or PE-labelled anti-mouse CD4 mAb (BioLegend, London, UK), and PE-labelled anti-mouse IL-4 and IL-17A (eBiocience Ltd., Hatfield, UK), or Pacific Blue^TM^ anti-mouse IL-2 (BioLegend, London, UK) antibodies (Abcam, Cambridge, UK) were used for flow cytometry. The CD4^+^ T cells from splenocytes of *B6;129S-Ldlr^tm1Her^Apob^tm2Sgy^/J* mice immunized with recombinant construct or control molecule Den were used for detection of the level of Foxp3 expression using a Treg detection kit (MACS, catalog no. 130-094-165), and the levels of IL-2, IL-4, and IL-17A population according to the manufacturer’s instructions.

For monocyte differentiation assay, mouse (C57BL/6) PBMCs were prepared using ficoll^®^ plaque plus (Sigma-Aldrich, Dorset, UK). In order to assess if serum of antigen-immunized mice could have effect on PBMC differentiation (by measuring the increased numbers of macrophages), antiserum of either AHHC or RHHC-immunized mice was pre-incubated with PBMCs before addition of antigen for stimulation. After 3 days, the expression of cell-surface marker CD206 (mannose receptor, macrophage marker) was assessed. Antigen- induced differentiation of monocytes into macrophages (as measured by flow cytometry) when compared to cell populations from non-induced cells as well as to control antigen (GST-den). Briefly, 3×10^5^ cells were incubated at 4°C for 30 min with appropriate dilutions of directly labeled monoclonal antibodies (allophycocyanin–anti-mouse CD206 antibody; BioLegend, Cambridge, UK). After 2 washing steps with 0.02% BSA/PBS (pH 7.3), fluorescence was analyzed on a flow cytometer (Cytomics FC500; Bachman coulter, High Wycombe, UK).

**Measurement of pro- and anti-inflammatory cytokines**

Levels of murine cytokines IL-10, TGF-β, TNF-α, and IFN-γ were measured in plasma using ELISA kits following the manufacturer’s instructions (R&D systems, Abingdon, UK). Levels of ConA-induced IL-10, TGF-β, TNF-α and IFN-γ in splenocyte cultures were measured. Briefly, 48-h spleen cell cultures of mice were stimulated with ConA (0, 1, 10,100 μg/ml). The cells were cultured for additional 24 h after adding ConA as stimulator. For antigen stimulation, splenocytes were cultured in RPMI 1640 with 10% fetal calf serum and induced with1 μg/ml antigen (GST-den, GST-AHHC, GST-RHHC, GST-RPHC, ApoB100 peptide, C5aR peptide, PAR-1 peptide, Cpn peptide, respectively) for 48 hour. Then IL10 and IFN-γ in supernatant of cultured cell were measured with DuoSet mouse IL-10 kit and mouse IFN-γ Quantikine immunoassay kit ( R&D system, Minneapolis) according to manufactory’s protocol.

**References**

- - - 1. Sutcliffe MJ, Jaseja M, Hyde EI, Lu X, Williams JA. Three-dimensional structure of the RGD-containing neurotoxin homologue dendroaspin. Nat Struct Biol. 1994;1: 802-807.
      2. Lu X, Xia M, Endresz V, Faludi I, Szabo A, Gonczol E, et al. Impact of multiple antigenic epitopes from Apob100, hHSP60 and *Chlamydophila* *pneumoniae* (*cpn*) on atherosclerotic lesion development in Apob^tm2sgy^Ldlr^tm1her^ J mice. Atherosclerosis. 2012;225: 56-68.
      3. Lu X, Rahman S, Kakkar VV, Authi KS. Substitutions of proline 42 to alanine and methionine 46 to asparagine around the RGD domain of the neurotoxin dendroaspin alter its preferential antagonism to that resembling the disintegrin elegantin. J Biol Chem. 1996;271: 289-294.
      4. Lu D, Chung KF, Xia M, Lu X, Scully M, Kakkar V. Integrin binding characteristics of the disintegrin-like domain of ADAM-15. Thromb Haemost. 2006;96: 642-651.
      5. Xia M, Chen D, Endresz V, Faludi I, Szabo A, Gonczol E, et al. Immunization of Chlamydia pneumoniae (Cpn)-Infected Apob(tm2Sgy)Ldlr(tm1Her)/J Mice with a Combined Peptide of Cpn Significantly Reduces Atherosclerotic Lesion development. PLoS One. 2013;8: e81056.
      6. Kilpatrick KE, Wring SA, Walker DH, Macklin MD, Payne JA, Su JL, et al. Rapid development of affinity matured monoclonal antibodies using RIMMS. Hybridoma. 1997;16: 381-389.
      7. Lu X, Chen D, Endresz V, Xia M, Faludi I, Burian K, et al. Immunization with a combination of ApoB and HSP60 epitopes significantly reduces early atherosclerotic lesion in Apobtm2SgyLdlrtm1Her/J mice. Atherosclerosis. 2010;212: 472-480.
